# In Situ Mass Spectrometry Diagnostics of Impaired Glucose Tolerance Using Label-Free Metabolomic Signature

**DOI:** 10.3390/diagnostics10121052

**Published:** 2020-12-05

**Authors:** Petr G. Lokhov, Oxana P. Trifonova, Dmitry L. Maslov, Elena E. Balashova

**Affiliations:** Institute of Biomedical Chemistry, 10 Building 8, Pogodinskaya Street, 119121 Moscow, Russia; oxana.trifonova@gmail.com (O.P.T.); dlmaslov@mail.ru (D.L.M.); balashlen@mail.com (E.E.B.)

**Keywords:** mass spectrometry, label-free, diagnostic signature, blood plasma, impaired glucose tolerance

## Abstract

In metabolomics, mass spectrometry is used to detect a large number of low-molecular substances in a single analysis. Such a capacity could have direct application in disease diagnostics. However, it is challenging because of the analysis complexity, and the search for a way to simplify it while maintaining the diagnostic capability is an urgent task. It has been proposed to use the metabolomic signature without complex data processing (mass peak detection, alignment, normalization, and identification of substances, as well as any complex statistical analysis) to make the analysis more simple and rapid. Methods: A label-free approach was implemented in the metabolomic signature, which makes the measurement of the actual or conditional concentrations unnecessary, uses only mass peak relations, and minimizes mass spectra processing. The approach was tested on the diagnosis of impaired glucose tolerance (IGT). Results: The label-free metabolic signature demonstrated a diagnostic accuracy for IGT equal to 88% (specificity 85%, sensitivity 90%, and area under receiver operating characteristic curve (AUC) of 0.91), which is considered to be a good quality for diagnostics. Conclusions: It is possible to compile label-free signatures for diseases that allow for diagnosing the disease in situ, i.e., right at the mass spectrometer without complex data processing. This achievement makes all mass spectrometers potentially versatile diagnostic devices and accelerates the introduction of metabolomics into medicine.

## 1. Introduction

Metabolomics has become a wide area of using “-omics” technologies for medical purposes, the results of which are promising for future implementation [1,2]. The Metabolomics Society states that it is worth noting that the study of metabolism at a global or “-omics” level is a fast-growing field that can have a profound impact on medical practice. Today, clinicians utilize only a very small part of the information contained in the metabolome, as they routinely measure only a narrow set of blood substances in order to assess health and disease states. It is expected that “the narrow range of chemical analyses in current use by the medical community today will be replaced in the future by analyses that reveal a far more comprehensive metabolic signature. This signature is expected to describe global biochemical aberrations that reflect patterns of variance in states of wellness, more accurately describe specific diseases and their progression, and greatly aid in differential diagnosis” [3].

**Figure 1:**
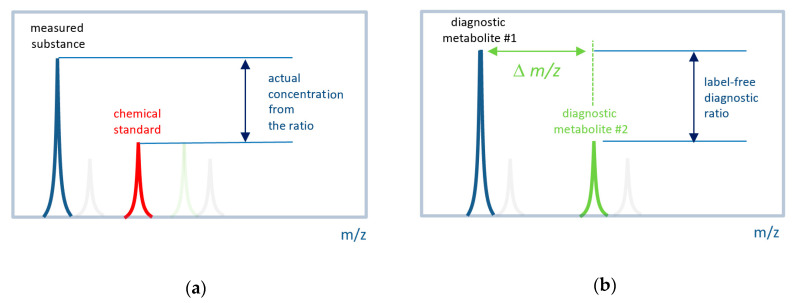
Concept of label-free in situ direct mass spectrometry-based diagnostics. (**a**) The conventional approach to measure the actual concentration of a substance by mass spectrometry. Labeled (e.g., by isotope) chemical standard is introduced into a sample with a known concentration. The ratio of a measured substance to its labeled standard is used to define the actual concentration. (**b**) The label-free approach uses signals (mass peak intensities) from two metabolites associated with the disease. It is expected that their ratio also has diagnostic power. A set of such ratios for many metabolites can be used to compile a diagnostic signature. The difference between the *m*/*z* (∆*m*/*z*) values must be small so that both peaks can be measured with the same instrumental parameters.

However, the complexity of metabolomics methods makes it difficult to introduce them into medicine, and the search for a way to simplify them while maintaining diagnostic capabilities is an urgent task. This study tested the concept of whether it is possible to use the technological performance of mass spectrometry, while reducing all of the data processing procedures up until the simple work of a mass spectrometer staff member at their workplace, i.e., in situ, using only a “pencil and a piece of paper”. To this end, the approach that takes place in vivo cytochrome P450 phenotyping [4] was used in the metabolomic signature generation [5]. In cytochrome phenotyping, instead of chemical standards application, the mutual ratio of the drug substance to its metabolite (the product of drug catabolism by cytochrome) is assessed a certain time after drug administration into the human body. Based on this, it can be assumed that metabolomic analysis, which is usually accompanied by complex analytical protocols and data processing (peak detection, alignment, normalization, deconvolution, identification, the usage of chemical standards, clusterization or complex decision-making procedures, etc.), can be simplified through the use of signatures with the ratios of the mass spectrometric peaks intensities. If the intensities of two mass spectrometric peaks are associated with the disease, their ratio may have diagnostic power (Figure 1), and a set of such ratios can be used to compile a diagnostic signature. The possibility of using such label-free signature was tested in this work for diagnosing impaired glucose tolerance (IGT). This widespread pre-diabetic state is associated with insulin resistance and an increased risk of cardiovascular pathology. Moreover, IGT has been shown to precede type 2 diabetes mellitus by many years [6]. Currently, the oral glucose tolerance test (OGTT) used for detecting IGT is time-consuming and may be dangerous for patients [7]. Therefore, a more rapid and patient-friendly test for diagnosing IGT is needed.

## 2. Materials and Methods

### 2.1. Blood Plasma Samples

The samples of blood plasma used in this study were taken from a previously published study [8]. Briefly, the blood samples for metabolomic analysis were taken from the vein before the morning meal. Samples (3 mL) were placed into glass tubes containing K_2_EDTA (BD Vacutainer; Becton, Dickinson and Company, Franklin Lakes, NJ, USA) and centrifuged within 15 min of blood collection at 1600× *g* and at room temperature. The resultant blood plasma was subdivided into aliquots that were pipetted into plastic tubes. These tubes were marked, transported in special thermo containers, frozen, and then stored at −80 °C until analysis. The analyzed samples were subjected to one freeze/thaw cycle. To test the reproducibility of this protocol, an additional set of blood samples (*n* = 20) was collected from the same individuals within 2–7 days of the original collection. For blood plasma deproteinization, sample aliquots (10 µL) were mixed with 10 µL of water (LiChrosolv; Merck KGaA, Darmstadt, Germany) and 80 µL of methanol (Fluka, Munich, Germany), and were incubated at room temperature. After 15 min, the samples were centrifuged at 13,000× *g* (MiniSpin plus centrifuge; Eppendorf AG, Hamburg, Germany) for 10 min. Deproteinized supernatants were then transferred to clean plastic Eppendorf tubes, and 50 volumes of methanol containing 0.1% formic acid (Fluka) were added to each tube. The resulting solutions were subjected to mass spectrometry analysis.

The metabolomics study of blood samples was approved by the relevant ethical review committee (the approval number is provided in the original study [8]). All of the procedures performed in studies involving human participants followed the ethical standards of the institutional or national research committee, and with the 1964 Helsinki Declaration and its later amendments or comparable ethical standards. Blood plasma concentrations of diagnostic substances (glucose, uric acid, total cholesterol, insulin, triglycerides, low-density lipoprotein (LDL), and high-density lipoprotein (HDL)) were measured using the Architect c4000 clinical chemistry analyzer (Abbott Diagnostics, Abbott Park, IL, USA). Glycated hemoglobin (HbA1c) was measured using the Bio-Rad D10 hemoglobin testing system (Bio-Rad Laboratories, Marne-la-Coquette, France). For the oral glucose tolerance test (OGTT), a standard glucose dose (75 g) was orally ingested and the blood glucose levels were checked 2 h later. IGT was diagnosed if the post-load glucose levels were between 7.8 and 11.0 mmol/l (WHO 1999) [9]. In this study, the OGTT results were used to establish the gender-matched cases (IGT; *n* = 20) and control (Normal; *n* = 20) groups. Table 1 presents the clinical characteristics of the cohort.

### 2.2. Mass Spectrometry Analysis

Samples were analyzed with the hybrid quadrupole time-of-flight mass spectrometer maXis impact (Bruker Daltonics, Billerica, MA, USA) equipped with an electrospray ionization (ESI) source. The mass spectrometer was set up to prioritize the detection of ions with a mass-to-charge ratio (*m*/*z*) ranging from 50 to 1000, scan (spectra) rate of 1 Hz, and a mass accuracy of 1–3 parts per million (ppm). The spectra were recorded in the positive ion charge detection mode. Samples were injected into the ESI source using a glass syringe (Hamilton Bonaduz AG, Bonaduz, Switzerland) connected to a syringe injection pump (KD Scientific, Holliston, MA, USA). The flow rate of samples to the ionization source was 180 µL/h, and samples were injected in a randomized order (e.g., control samples were run between case samples). Mass spectra were obtained using DataAnalysis version 3.4 (Bruker Daltonics) to summarize the 1-min signals. Ion metabolite masses were determined from the mass spectrum peaks obtained using the DataAnalysis program. All of the peaks above noise level (signal to noise ratio >1) were selected, and the metabolite ion masses were pooled and processed using Matlab version R2010a (MathWorks, Natick, MA, USA). The alignment of the mass peaks was performed as described previously [10]. Mass peak intensities were normalized as described previously [11]. All calculations were performed using Matlab software.

### 2.3. Compilation of Label-Free Diagnostic Signature

**Figure 2:**
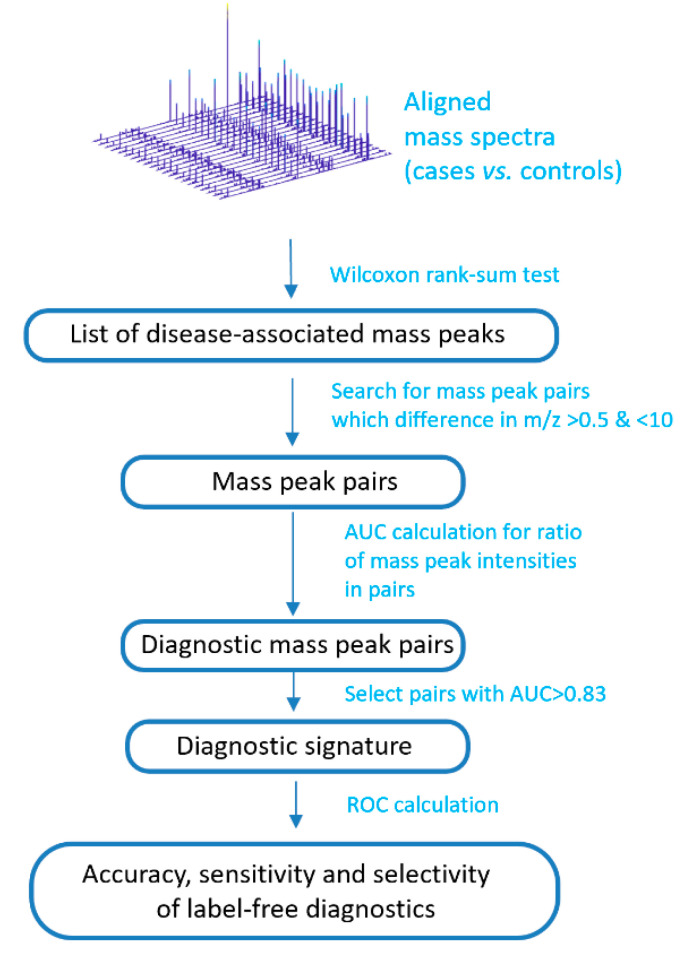
Schema to compile a label-free diagnostic signature based on direct-infusion mass spectrometry data. ROC: receiver operating characteristic; AUC: area under ROC curve.

The peak intensities of the metabolites associated with IGT were included in the calculation of a mass spectrometry signature (Figure 2). To this end, mass spectrometry peaks with intensities associated with an IGT of *p* < 0.01 were selected (calculated by the Wilcoxon rank-sum test; *ranksum* function in Matlab). Furthermore, the signature search algorithm formed pairs using mass spectrometry peaks with an *m*/*z* difference of 0.5 to 10. The ratio of intensities in each pair was calculated, while only the peaks presented in all of the mass spectra of the samples were used. The ratio was calculated by dividing the intensity values of the more intense peak by the less intense, which were determined from their mean values for the set of the mass spectra. For the obtained ratios, the ROC curves were generated using the *perfcurve* function of the Matlab program. This function presents the accuracy, sensitivity, and specificity for each point of ROC curve, and area under ROC curve (AUC). To compile a diagnostic signature, ratios with AUCs above the optimal threshold were taken. To find the optimal threshold, this threshold was changed stepwise, and the number of metabolite ions included in the signature and the value of the diagnostic scores (see next section) were evaluated, as well as the corresponding diagnostic accuracy, sensitivity, and specificity. Thus, the most optimal signature was chosen, which shows a high diagnostic accuracy, but at the same time, the number of included mass peak pairs is not very large, which allows for performing diagnostics on site.

### 2.4. Diagnostic Score Calculation

The diagnostic score was calculated as previously described [8], but adapted to the ratio of the mass spectrometric peaks used in the signature. If the ratio in the pair included in the signature was higher than the threshold value, then the diagnostic score was increased by one. The diagnostic scores obtained in this way for all samples were used to generate the ROC curve and to determine the threshold value of the diagnostic score, the excess of which indicates the presence of IGT. For the defined threshold value for the diagnostic score, the AUC, diagnostic accuracy, specificity, and sensitivity were calculated. The *perfcurve* function was also used for this.

### 2.5. Leave-One-Out Testing

The diagnostic signature was additionally validated using the leave-one-out method [12]. This method involves the one-by-one removal of each data point (sample) from the dataset and rebuilding the diagnostic signature based on the remaining data. The rebuild signature was then tested by the excluded sample. Thus, the diagnostic signature was tested for 40 samples.

## 3. Results

**Figure 3:**
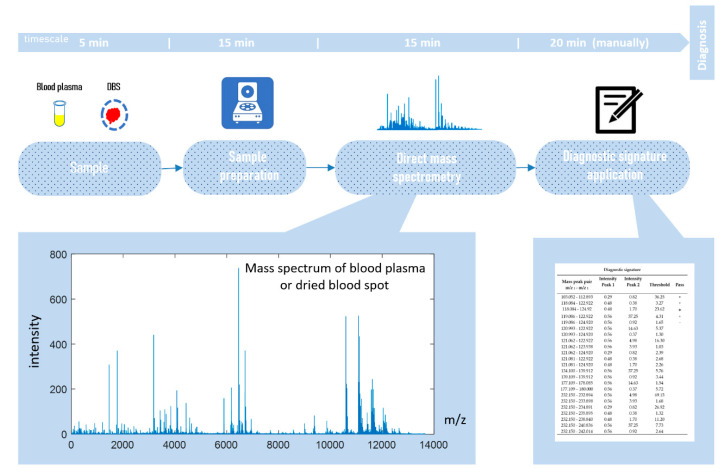
Workflow for in situ mass spectrometry diagnostics.

**Figure 4:**
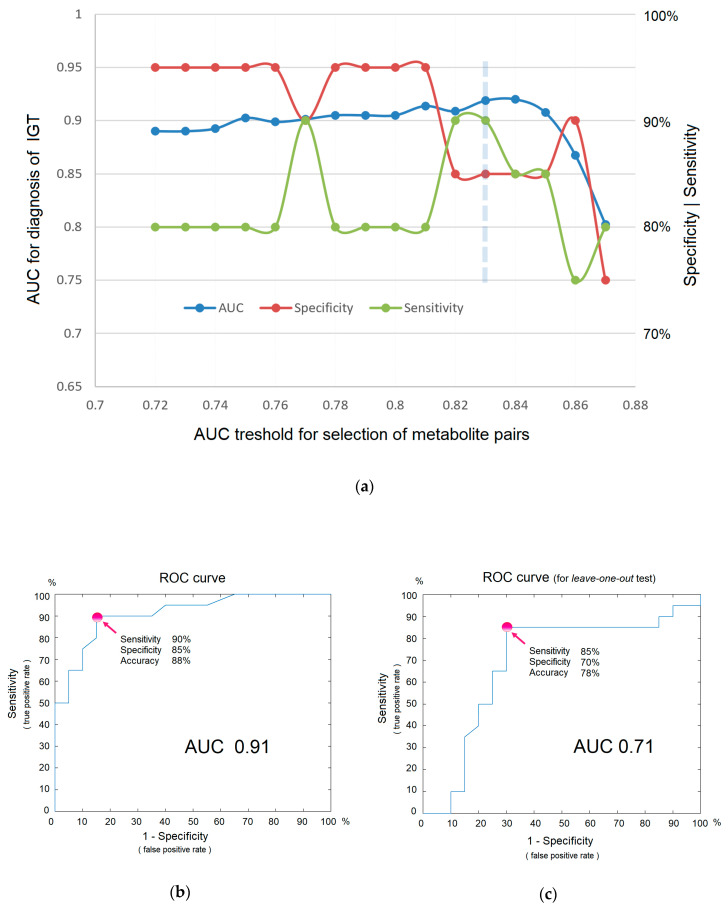
The efficiency of in situ mass spectrometric diagnostics without labels. (**a**) Dependence of diagnostic parameters on metabolites included in the signature (i.e., on the AUC threshold for the ratio of mass peak intensities, as a criterion for inclusion in the signature). The optimal threshold is shown by the dashed line. (**b**) ROC curve for IGT diagnosis. (**c**) ROC curve for IGT diagnostics obtained using the leave-one-out test. Arrows indicate points on the ROC curve corresponding to optimal sensitivity, specificity, and accuracy of diagnostics.

Direct-infusion mass spectrometry analysis of plasma samples resulted in the detection of about 4000 low weight molecular ions per sample (Figure 3). The total analysis time for one sample was 30 min, which could be sufficiently decreased by sample preparation in parallel mode. The application of the diagnostic signature search algorithm resulted in the detection of numerous mass peak pairs, with the diagnostic power revealed by the AUC calculation. To select the peak pairs for the diagnostic signature, with AUC threshold value was increased stepwise (Figure 4a) for the ratio of the peak intensities, which resulted in the selection of pairs with AUC >0.83 for the final (optimal) diagnostic signature. This signature showed a diagnostic accuracy 88% (AUC 0.91, specificity 85%, and sensitivity 90%; Figure 4b). A diagnostic score of 22 units was identified as the threshold value for distinguishing IGT versus normal states.

Although the obtained signature is not a result of a training algorithm, where a small number of samples with an excessive number of variables leads to overfitting and requires testing on samples that are not involved in building a diagnostic model, the leave-one-out testing was nevertheless performed. The reason for this is that multiple measurements also lead to a distortion of diagnostic accuracy. The test result showed AUC 0.71, specificity 0.70, and sensitivity 0.85 (Figure 4c), which, as it was expected, are lower than in the original model. Given that AUC values ranging from 0.5–0.6 indicate that a test does not work; 0.6–0.7 is a poor, yet functional, test; 0.7–0.8 is a good test; and 0.9–1.0 is an excellent test [13], it can be concluded that the concept of in situ diagnostics has been confirmed and could be regarded as a good test using the example of IGT diagnostics.

Table 2 demonstrates that the data related to the diagnostic signature consisted of 45 metabolite ion peaks, for which relations are used to calculate the diagnostic score for IGT.

## 4. Discussion

The implementation of omics tests that allow for multiple measurements of substances in one analysis is an applied direction in the omics sciences. The idea of using complex but efficient methods of analysis in medical practice is a trend and, to a certain extent, is supervised by the committee, some aspects of which are presented in the book “Evolution of Translational Omics: Lessons Learned and the Path Forward” [14]. Among the possible omiсs tests, metabolomics tests are the most promising. The continuous improvement of metabolomics methods points to their long-overdue introduction into medicine, and, according to the declaration of the Metabolomic Society, metabolomics should replace separate clinical laboratory tests in the future [3]. However, this process is extremely difficult. The reason for this is quite simple; the big data generated by omics tests require complex processing, which is far from trivial, and turns the metabolomic analysis into a complex scientific work that does not fit a routine clinical laboratory test in terms of time, work, and cost. Earlier, there were attempts to create metabolomic signatures for clinical use [5,15,16,17,18], but that was not enough.

The literature review shows that successful experience in the use of mass spectrometry for in vivo cytochromes P450 phenotyping by drugs can significantly simplify the metabolomics diagnostics through the use of metabolomic signatures. During phenotyping, the decrease in drug concentration as well as the increase in its metabolite are informative, so the use of their ratios is justified and sufficiently simplifies the phenotyping procedure. The use in signatures for the ratios of mass spectrometric peaks, which are close to each other in the mass spectrum, eliminates the need for complex data processing. The intensity of such peaks is equally modulated by the instrumental measurement parameters, and therefore excessive data processing is not required. This work, using the example of IGT diagnostics, has confirmed the possibility of creating such diagnostic signatures with a good diagnostic power (i.e., with AUC >0.7).

The study of in situ diagnostics using direct mass spectrometry is not accidental. It has been successfully used to study cancer [10,19], diabetes [8], heart disease [20], obesity [11], Parkinson’s disease [21], and Alzheimer’s disease [22]. Direct mass spectrometry is characterized by a high processing speed and high reproducibility of data, which are useful for clinical purposes [23,24,25]. However, more importantly, direct mass spectrometry provides the metabolomic profile of the sample per se, without the distortion introduced by the separation method, be it liquid or gas chromatography. As the signature uses the ratio of nearby substances in the spectrum, the use of chromatography is unacceptable. The substances, which help to form diagnostic pairs, can be eluted from the column at different times, and therefore their ratio cannot be evaluated simultaneously at a glance for diagnostics in situ. Moreover, coeluted substances, as a result of ion suppression, can influence their intensities, and the diagnostic efficacy depend on the chromatographic conditions. This makes the use of hyphenated techniques for in situ diagnosis impractical.

The choice of IGT for testing the new metabolomic signature had its reasons. Currently, OGTT represents the “gold standard” for detecting IGT. However, OGTT is time-consuming (takes 2 h) and some people may experience sugar shock during it [7]. Therefore, a more rapid test for diagnosing IGT is needed, and the development of in situ diagnostics is necessary. Moreover, earlier, the diagnostic metabolomic signature for IGT was published [8], which can be used to compare the effectiveness of the new signature. The new signature demonstrated a diagnostic accuracy of 88%, while the previously published signature achieved 90%, indicating an insignificant decrease in the diagnostic efficiency with a significant simplification of the diagnostic procedure.

Although metabolite identification may provide more information about the status of the disease, within the framework of this study, the identification of metabolites forming the signature was not carried out, as this is outside the concept of in situ diagnostics. This diagnostic uses the full power of mass spectrometric profiling, where the number of detected substances are an order of magnitude more than it is possible to identify. Nevertheless, it can be noted that ^13^C isotopes of the same substances are presented in the signature. The isotopes were not deliberately removed from the signature, thereby emphasizing the automatic formation of the signature. Such an automatic approach (without any manual correction) allows for concluding the possibility of creating in situ diagnostics for other diseases according to the described model.

## 5. Conclusions

The concept of an in situ diagnosis of disease has been successfully tested using the example of IGT. This diagnostic allows for the use of mass spectrometers directly in order to analyze the biological material and make a diagnosis in situ in the workplace in a matter of minutes. Thus, mass spectrometers can be considered effective diagnostic devices. Moreover, such a diagnostic is universal (one spectrum, many signatures), and is significantly better in terms of time and cost than the available clinical tests. The demonstrated approach for generating label-free diagnostic signatures can be recommended for creating in situ diagnostics for other diseases, thereby accelerating the introduction of highly effective metabolomic methods into clinical practice.

## Figures and Tables

**Table 1:** Clinical characteristics of the patient cohort.

Characteristics	Value		*t*-Test (*p*-Value)	AUC
	Control Subjects	Subjects with IGT		
Number	20	20	N/A	N/A
Sex (male/female)	10/10	10/10	1	0.50
Age (years)	56.1 ± 13.9	61.1 ± 10.2	0.21	0.59
BMI (kg/m^2^)	36.1 ± 9.1	33.7 ± 7.6	0.38	0.47
Glucose in OGTT (mmol/L)	6.4 ± 1.0	10.6 ± 1.7	0	1.00 ^1^
Fasting glucose (mmol/L)	5.3 ± 0.3	5.5 ± 0.3	0.04	0.66
HbA1c (%)	5.7 ± 0.4	6.1 ± 0.4	0.0032	0.74
Insulin (µU/mL)	11.3 ± 7.9	12.8 ± 6.3	0.52	0.62
Cholesterol (mmol/L)	5.2 ± 0.8	4.9 ± 1.2	0.39	0.43
Uric acid (µmol/L)	389 ± 86	382 ± 84	0.81	0.48
HDL (mmol/L)	1.2 ± 0.4	1.1 ± 0.4	0.38	0.42
LDL (mmol/L)	3.5 ± 0.8	3.0 ± 0.9	0.1	0.35
Triglycerides (mmol/L)	1.3 ± 0.5	2.2 ± 2.7	0.15	0.66
HOMA-β	126 ± 87	129 ± 73	0.8	0.58
HOMA-IR	2.7 ± 1.9	3.1 ± 1.7	0.43	0.61

The AUC for glucose (OGTT) is equal to 1, as the OGTT test was used to establish the control and impaired glucose tolerance (IGT) groups. AUC—area under ROC curve; ROC—receiver operating characteristic; OGTT—oral glucose tolerance test; HOMA—homeostatic model assessment; BMI—body mass index; N/A—not applicable.

**Table 2:** Data related to the label-free diagnostic signatures.

Mass Peak Pair *m*/*z*_1_–*m*/*z*_2_	Mean		Wilcoxon Rank-Sum Test (*p*-Value)	Threshold	AUC	Specificity	Sensitivity
	For *m*/*z*_1_	For *m*/*z*_2_					
103.052–112.893	1.75	59.39	0.0002	36.25	0.84	0.90	0.75
118.084–122.922	20.40	7.53	0.0002	3.27	0.85	0.85	0.75
118.084–124.92	20.40	1.13	0.0001	23.62	0.86	0.95	0.65
119.086–122.922	1.37	7.53	0.0001	4.31	0.86	0.70	0.90
119.086–124.920	1.37	1.13	0.0002	1.65	0.85	1.00	0.60
120.993–122.922	1.60	7.53	0.0003	5.37	0.83	0.80	0.80
120.993–124.920	1.60	1.13	0.0002	1.30	0.84	0.80	0.80
121.062–122.922	0.44	7.53	0.0004	16.30	0.83	0.75	0.90
121.062–123.938	0.44	0.47	0.0002	1.03	0.85	0.80	0.90
121.062–124.920	0.44	1.13	0.0004	2.39	0.83	0.75	0.90
121.081–122.922	2.15	7.53	0.0002	2.68	0.85	0.70	0.95
121.081–124.920	2.15	1.13	0.0002	2.26	0.85	0.80	0.80
134.100–139.912	0.14	0.82	0.0002	5.76	0.84	0.70	0.90
139.109–139.912	0.29	0.82	0.0002	3.44	0.85	0.90	0.75
177.109–178.085	0.48	0.38	0.0002	1.54	0.85	0.85	0.90
177.109–180.000	0.48	1.70	0.0001	5.72	0.86	0.90	0.80
232.150–232.894	0.56	37.25	0.0002	69.15	0.85	0.80	0.75
232.150–233.898	0.56	0.92	0.0001	1.68	0.86	0.80	0.75
232.150–234.891	0.56	14.63	0.0002	26.92	0.85	0.80	0.80
232.150–235.895	0.56	0.37	0.0002	1.32	0.84	0.65	0.90
232.150–238.840	0.56	4.98	0.0001	11.20	0.86	0.90	0.65
232.150–240.836	0.56	3.93	0.0001	7.73	0.86	0.80	0.75
232.150–242.014	0.56	1.07	0.00004	2.64	0.88	1.00	0.60
233.148–242.923	1.67	22.86	0.0002	14.18	0.84	0.80	0.80
234.079–242.923	0.21	22.86	0.0003	135.09	0.83	1.00	0.55
234.079–243.927	0.21	0.87	0.0003	4.04	0.83	0.70	0.85
234.151–242.923	0.27	22.86	0.0002	95.66	0.85	0.90	0.700
234.151–243.927	0.27	0.87	0.0001	3.13	0.86	0.70	0.90
239.013–240.836	0.28	3.93	0.0003	12.74	0.84	0.75	0.85
241.965–244.921	0.22	1.84	0.0002	10.37	0.85	1.00	0.50
246.047–248.868	0.22	16.81	0.0003	78.80	0.83	0.85	0.80
247.165–248.868	0.50	16.81	0.0003	30.40	0.84	0.75	0.90
247.165–249.872	0.50	0.42	0.0003	1.31	0.83	0.90	0.80
255.116–258.896	1.37	6.26	0.0003	5.41	0.84	0.90	0.75
256.153–261.920	1.70	0.37	0.0003	4.73	0.83	0.80	0.75
262.012–264.844	0.31	7.14	0.0002	20.91	0.84	0.75	0.90
262.012–266.968	0.31	0.32	0.0001	1.08	0.88	0.85	0.80
295.935–301.958	0.50	0.59	0.0003	1.11	0.84	0.65	0.90
